# Abnormality of m6A mRNA Methylation Is Involved in Alzheimer’s Disease

**DOI:** 10.3389/fnins.2020.00098

**Published:** 2020-02-28

**Authors:** Min Han, Zhen Liu, Yingying Xu, Xiangtian Liu, Dewei Wang, Fan Li, Yun Wang, Jianzhong Bi

**Affiliations:** ^1^Department of General Medicine, The Second Hospital of Shandong University, Jinan, China; ^2^Department of Neurology Medicine, Second Hospital of Shandong University, Jinan, China; ^3^Medicine School, Shandong University, Jinan, China

**Keywords:** N6-methyladenosine (m6A), AD, synapse, methyltransferase, demethylase, METTL3, FTO

## Abstract

Alzheimer’s disease (AD), the most common form of dementia, is highly prevalent in older adults. The main clinical feature is the progressive decline of memory function, which eventually leads to the decline of cognitive function. At present, the pathogenesis of AD is unclear. In the disease process, synaptic changes are the key. Recent studies have shown that the dysregulation of RNA methylation is related to many biological processes, including neurodevelopment and neurodegenerative diseases. N6-methyladenosine (m6A) is the most abundant modification in eukaryotic RNA. In this study, RNA m6A methylation was quantified in APP/PS1 transgenic mice, which is an AD mouse model, and C57BL/6 control mice, and data showed that m6A methylation was elevated in the cortex and the hippocampus of APP/PS1 transgenic mice. Next, the alterations of m6A RNA methylation in AD and in C57BL/6 mice were investigated using high-throughput sequencing. Genome-wide maps of m6A mRNA showed that the degrees of m6A methylation were higher in many genes and lower in others in AD mice. Interestingly, the expression of the m6A methyltransferase METTL3 was elevated and that of the m6A demethylase FTO was decreased in AD mice. The data were analyzed by gene ontology (GO) and Kyoto Encyclopedia of Genes and Genomes (KEGG) pathway analyses, and pathways that might be related to synaptic or neuron development and growth were constructed. The related pathways and genes predicted the potential roles of the differentially expressed m6A methylation RNA in AD. Collectively, our findings demonstrate that the m6A methylation of RNA promotes the development of AD.

## Introduction

The incidence of Alzheimer’s disease (AD) in many countries around the world is very high due to a lack of effective treatments (Bateman et al., 2012). Many reports attempt to detail the pathogenesis of AD; however, the subject is still debated and no definite conclusion has been reached, potentially because there is no single AD pathogenesis. At present, researchers agree that changes in synaptic function are involved in the pathogenesis of AD, including the dysfunction and loss of the synapse (Mucke and Selkoe, 2012); however, the specific molecular mechanism still remains unresolved, and thus the pathogenesis of AD is still unknown.

The brain is very rich in N6-methyladenosine (m6A). Some studies have shown that m6A is related to the development of the nervous system and to neural degenerative diseases (Meyer et al., 2012; Widagdo et al., 2016; Li et al., 2018), but the role of the regulatory mechanism is still unknown. m6A is a biological marker of dynamic and reversible regulation (Niu et al., 2013), which relies on the combined action of methyltransferase and demethylase. Currently, known methyltransferases include methyltransferase-like protein 3 (METTL3), METTL14, and Wilms tumor 1-associating protein (Liu et al., 2014; Wang and Zhao, 2016; Vu et al., 2017), while demethylases include AlkB homolog 5 and obesity-associated protein (FTO) (Zhou et al., 2015). Among them, METTL3 is the at the center of catalytic methyl reactions, and FTO, which is abundant in rat brains, is related to neurotransmitter delivery and nervous system development (Li et al., 2017, 2018). m6A RNA methylation, regarded as a new frontier in neuroscience, could provide us with a better understanding of neural development and neurological diseases from a novel perspective. In this study, m6A RNA methylation in the brains of AD and of control mice were investigated by high-throughput sequencing and the differences were compared. Further, gene ontology (GO) and Kyoto Encyclopedia of Genes and Genomes (KEGG) pathway analyses were used to predict the function of differentially expressed RNAs.

## Materials and Methods

### Mouse Models

All mice used in this study were male (*n* = 10 per group). Heterozygous double-transgenic human mice (APP/PS1) at 9 months of age were used as a model for AD, and age-matched C57BL/6 mice were used as controls. All mice were purchased from Beijing HFK Bio-Technology Co., Beijing, China. The mice were housed at 25 ± 2°C in controlled rooms. After 2 weeks, the mice were euthanized using 10% chloral hydrate, and their cerebral cortex, hippocampus, and cerebellum were dissected. All procedures were carried out under the guidelines of the Ethical Committee for Animal Experiments of Shandong University (Jinan, China). Using liquid nitrogen, tissues were immediately frozen after dissection and stored at −80°C until analysis.

### RNA Isolation

From frozen mouse cerebral cortex, hippocampus, and cerebellum sections, total RNA was purified using TRI-Reagent (Cat. No.15596026, Thermo Fisher Scientific). The quality of RNA was analyzed using a DeNovix spectrophotometer, and samples with A260/A280 ratios between 1.9 and 2.2 were used for further experiments.

### High-Throughput Sequencing

High-throughput sequencing was completed by Shanghai Cloud-seq Biotech Co., Ltd., using mouse hippocampus samples (*n* = 3 per group). The analysis screened for genes that differentially expressed m6A methylation when comparing the AD and the control groups. GO and KEGG analyses were performed to see if these genes had different physiological functions. *P* < 0.05 was considered as statistically significant.

### Quantification of the m6A Modification

The change in global m6A levels in total RNA was measured using an m6A RNA Methylation Quantification Kit (colorimetric; Abcam, ab185912) according to the manufacturer’s protocol. For each sample analysis, 200 ng of total RNA was used. The absorbance was measured on a microplate reader at 450 nm, and the m6A horizontal colorimetric value was measured according to the standard curve.

### Quantitative Real-Time PCR

One microgram of total RNA from mouse hippocampus samples was used to synthesize cDNA using the PrimeScript First Strand cDNA Synthesis Kit (Takara, RR047A). The cDNA was analyzed to determine the relative RNA levels of target genes by quantitative real-time PCR (qRT-PCR) with the SYBR Green detection method (Takara, RR041A) using the StepOnePlus Real-time PCR System (Eppendorf, Mastercycler, Germany). The procedure was 40 cycles of 95°C for 30 s, 95°C for 15 s, and 55°C for 15 s, and β-actin was used as a normalization control. Genes targeted in qRT-PCR were selected based on the results of the high-throughput sequencing analysis, including two genes with increased methylation in the AD group compared with the control group (AMPA and NMDA) and one with decreased methylation in the AD group (SEMA). The expression levels of METLL3 and FTO were also verified. The following primers were used:

Mouse METTL3 forward: 5′-TTAGCATCTGGTCTGGC CTCTT-3′
Mouse METTL3 reverse: 5′-TGACCTTCTTGCTCTG CTGTTC-3′
Mouse FTO forward: 5′-GACACTTGGCTTCCTT ACCTG-3′
Mouse FTO reverse: 5′-CTCACCACGTCCCGAA ACAA-3′
Mouse AMPA forward: 5′-GGGACAACTCAAGCG TCCAGA-3′
Mouse AMPA reverse: 5′-GCAGCCAGTTCCACG CAGTA-3′
Mouse NMDA forward: 5′-GGCTGACTACCCGAAT GTCCA-3′
Mouse NMDA reverse: 5′-TGTAGACGCGCATCATCT CAAAC-3′
Mouse SEMA forward: 5′-ACAGCTCCAGTTACCACA CCTTC-3′
Mouse SEMA reverse: 5′-TGTAGACGCGCATCATC TCAAAC-3′
Mouse β-actin forward: 5′-CATCCGTAAAGACCTCTAT GCCAAC-3′
Mouse β-actin reverse: 5′-CCCAGTTCCACAGGC ATACA-3′

The real-time PCR reactions were performed in triplicate, and the results were analyzed using the ΔΔCT method (Livak and Schmittgen, 2001).

### Western Blot

The cerebral cortex, cerebellum, and hippocampus tissues were stored at −80°C until homogenization and were then used to detect m6A RNA methylase and demethylase. To assess the quantifiable factors of neurodegenerative processes and amyloid burden in these animals, cortical and hippocampal brain tissues were used to measure the expression of synaptophysin and Aβ protein in both groups of mice. The samples were homogenized separately in ice-cold RIPA lysing buffer for 30 min and then were centrifuged at 12,000 × *g* for 15 min at 4°C. Protein concentrations were detected using the bicinchoninic acid method. Briefly, the proteins were separated using SDS-PAGE and transferred to PVDF membranes. Then, the membranes were pretreated with a blocking solution (5% non-fat dry milk) for 1 h and incubated with primary antibodies against METTL3, FTO, beta amyloid, synaptophysin, and β-actin overnight at 4°C. After rinsing with a washing solution (TBST) three times, the membranes were incubated with secondary antibodies for 1 h and washed three times with TBST. The intensity of the protein bands was analyzed using Image J (National Institutes of Health, Bethesda, MD, United States).

The following reagents and antibodies were used: RIPA lysis buffer (Beyotime Biotechnology, P0013B), primary antibody dilution buffer (Beyotime Biotechnology, P0256), protease and phosphatase inhibitor (Beyotime Biotechnology, P1050), anti-METTL3 rabbit IgG (Abcam, ab195352), anti-FTO rabbit IgG (CST, 45980), anti-beta amyloid rabbit polyclone (Abcam, ab2539), anti-synaptophysin rabbit IgG (Abcam, ab32137), anti-actin (CST, 4970), and HRP-labeled goat anti-rabbit IgG secondary antibody (Beyotime Biotechnology, A0208).

### Statistical Analysis

All values are expressed as means ± SEM of at least three independent experiments. Student’s *t*-test was performed to identify statistical differences among groups. *P* < 0.05 was considered as statistically significant. SPSS17.0 software (SPSS Inc., Chicago, IL, United States) was used for data analysis.

## Results

### m6A RNA Methylation Level in the Cortex and the Hippocampus of AD

**FIGURE 1:**
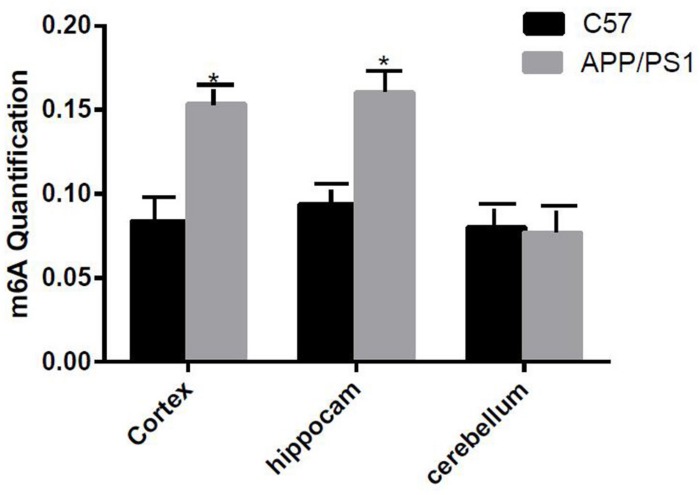
Comparison of methylation content of the cortex, hippocampus, and cerebellum samples between the two groups. Among them, m6A RNA methylation significantly increased in the hippocampus and cortex samples. Student’s *t*-test was used to detect differences between the two groups, and data are presented as mean ± SEM; *n* = 6 per group, *P < 0.05.

The degree of methylation in the cortex and the hippocampus samples of AD mice were notably higher than that of the control group (*P* < 0.05); however, no difference in the degree of methylation was observed in the cerebellum (Figure 1).

### GO Analysis and KEGG Pathway Analysis of Differentially Expressed m6A RNA Methylation-Derived Genes

**FIGURE 2:**
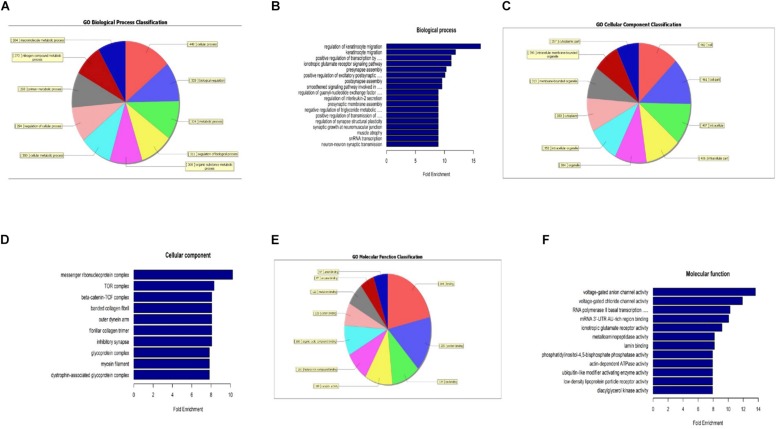
Gene ontology analysis showing that m6A RNA methylation is related to specific functional roles in AD. The analyzed genes with increased m6A RNA methylation expression in the AD group are shown; biological process-specific function **(A,B)**, cell component-specific function **(C,D)**, and molecular function-specific function **(E,F)**.

**FIGURE 3:**
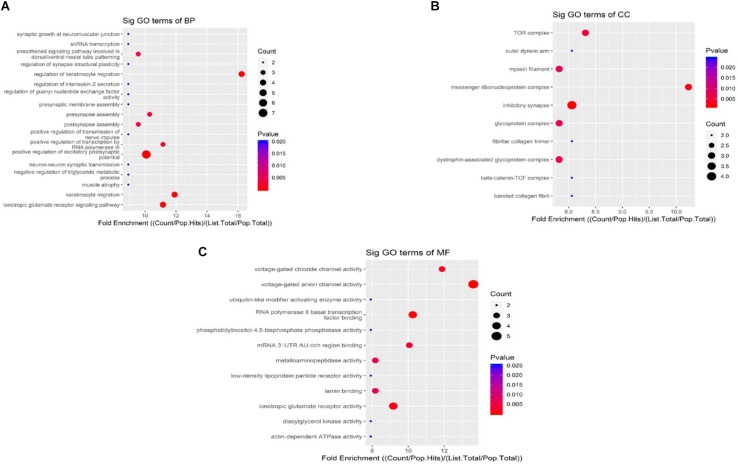
Kyoto Encyclopedia of Genes and Genomes pathway analysis of genes with differentially expressed m6A RNA methylation, showing the top 18 enrichment scores. Analysis predicted the pathways affected by the variation of mRNAs in AD brain samples; biological processes **(A)**, cell components **(B)**, and molecular functions (**C**) show their own pathways. The enrichment score was calculated as –log10 (*P*-value).

**FIGURE 4:**
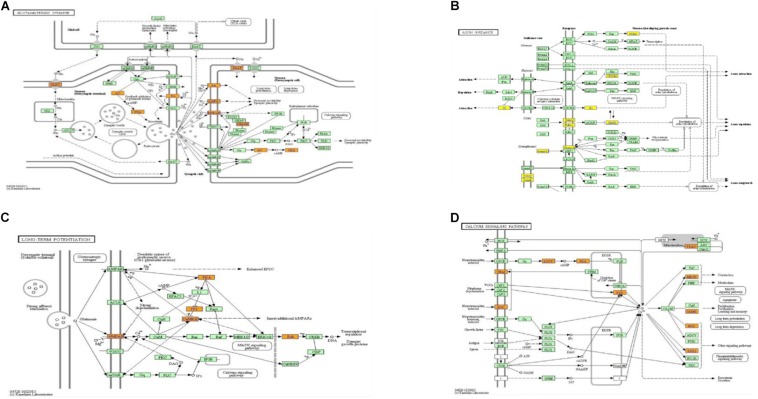
Pathways associated with synaptic function, from which genes to be studied were selected. Glutamatergic synapse **(A)**, axon guidance **(B)**, long-term potentiation **(C)**, and calcium signaling pathway **(D)**.

When compared to the control group, the degree of methylation in the AD group increased in 659 genes, while the degree of methylation decreased in 991 genes. In order to clarify if m6A RNA methylation was related to specific functional roles in AD, GO term enrichment analyses of target RNAs identified in the AD and in the control groups were performed, revealing increased methylation of m6A in the AD group compared to that in the control group. GO analysis consisted of three parts: biological processes (BP), cell components (CC), and molecular functions (MF). The GO analysis of BP showed that the genes with increased expression of m6A RNA methylation in the AD group were significantly associated with the regulation of dendrite development, transport, positive regulation of cellular components and processes, cellular component organization or biogenesis, positive regulation of BP, nervous system development, and single-organism processes. Significant GO CC terms of differentially expressed RNAs showed that these mRNAs were associated with the cytoplasm, organelles, cell parts, intracellular parts, and intracellular organelles. For MF, these mRNAs were associated with the binding of specific substances, such as proteins, enzymes, metal ions, cations, and heterocyclic compounds, and catalytic activity (Figures 2A–F). The KEGG pathway dot-plot revealed the significant enrichment pathways with the top 18 enrichment scores [−log10 (*P*-value)]. KEGG pathway analysis predicted the pathways affected by the variation of mRNAs in the AD brain samples, including synaptic growth at the neuromuscular junction, snRNA transcription, the smoothened signaling pathway involved in dorsal/ventral neural tube patterning, regulation of synapse structural plasticity, regulation of keratinocyte migration, regulation of interleukin-2 secretion, regulation of guanyl-nucleotide exchange factor activity, presynaptic membrane assembly, pre-synapse assembly, post-synapse assembly, positive regulation of transmission of nerve impulse, positive regulation of transcription by RNA polymerase III, positive regulation of excitatory post-synaptic potential, neuron–neuron synaptic transmission, negative regulation of triglyceride metabolic process, muscle atrophy, keratinocyte migration, and ionotropic glutamate receptor signaling pathway (Figures 3A–C). Additionally, the four main pathways were associated with synapses, glutamatergic synapse, axon guidance, long-term potentiation, and calcium signaling pathway (Figures 4A–D).

### Ubiquitous Expression of m6A Methyltransferases and Demethylases Between the Two Groups

**FIGURE 5:**
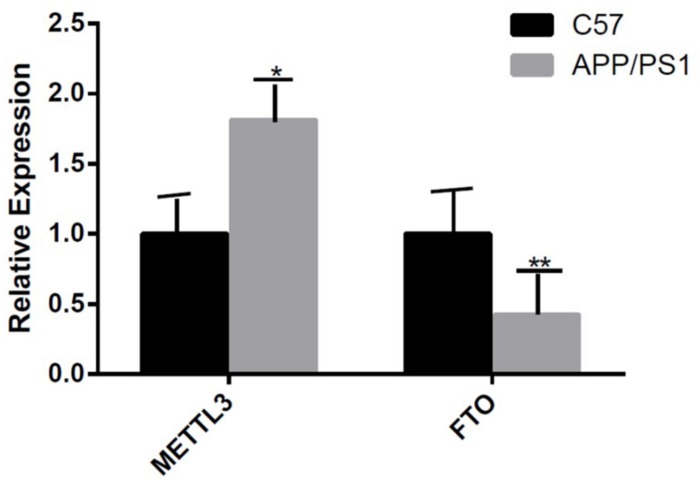
The expression of METTL3 gene is increased and that of FTO gene is decreased in the AD group. Student’s *t*-test was used to detect differences between the two groups, and data are presented as mean ± SEM; *n* = 5 per group, *P < 0.05, **P < 0.01.

**FIGURE 6:**
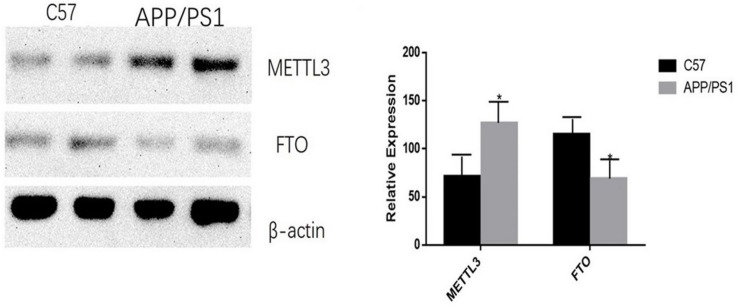
AD group transplantation versus the control group. Expression of METTL3 and FTO enzymes in the brains of AD mice is different from that in the control group. The relative densities of all proteins bands are normalized to β-actin. Student’s *t*-test was used to detect differences between the two groups, and data are presented as mean ± SEM; *n* = 6 per group, *P < 0.05.

The mRNA expression of METTL3 and FTO was examined. The results showed an increased expression of METTL3 (*P* < 0.05) and a decreased expression of FTO genes in the AD group (*P* < 0.01; Figure 5). Further, compared to the control group, the expression of METTL3 in the cortex and the hippocampus in AD mice was significantly higher (*P* < 0.05). However, no significant difference in the expression of METTL3 in the cerebellum was found between the two groups. Additionally, the level of the demethylase FTO in the hippocampus of AD mice was lower than that of control mice (*P* < 0.05), while no significant differences were observed in its expression in the cortex and the cerebellum (Figure 6).

### The Levels of Aβ Deposition and Synaptophysin Expression Were Different in the Two Groups of Mice

**FIGURE 7:**
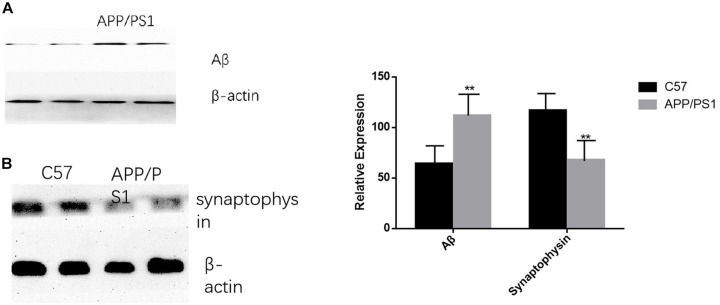
AD animal model validation results. **(A)** The expression level of Aβ in APP/PS1 mice is significantly increased compared with that of the control group, while synaptophysin is decreased in AD animal models **(B)**. Student’s *t*-test was used to detect differences between the two groups, and data are presented as mean ± SEM; *n* = 6 per group, **P < 0.01.

The deposition of Aβ was significantly increased in AD mice, while the opposite trend occurred for synaptophysin (*P* < 0.01). The results were consistent between the cortex and the hippocampus (Figures 7A,B).

### At Different Levels of m6A RNA, Methylation and Quantity of Gene Expression Were Different

**FIGURE 8:**
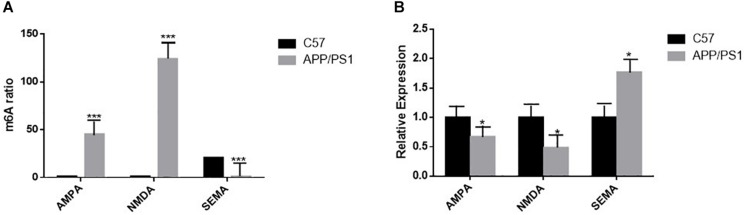
**(A)** High-throughput sequencing analysis shows statistically significant differences in m6A RNA methylation of AMPA, NMDA, and SEMA in the hippocampus of the two groups of mice; *n* = 3 per group, ***P < 0.001. **(B)** The mRNA levels of AMPA and NMDA in the brains of AD mice were reduced by quantitative RT-PCR, and that of SEMA is the opposite. β-actin was measured as the reference gene; *n* = 5 per group. Student’s *t*-test was used to detect differences between the two groups; data are presented as mean ± SEM; *P < 0.05.

The results of high-throughput sequencing analysis showed that the methylation degree of AMPA was increased in the AD group compared to that in the control group, and its gene expression by qRT-PCR was decreased in the AD group. The trend of NMDA was the same as that of AMPA; however, in the AD group, the level of SEMA gene methylation decreased, and the amount of gene expression by qRT-PCR increased (Figures 8A,B).

## Discussion

APP/PS1 double transgenic mouse models are often used as a simulation of AD animal models. The advantage lies in the combination of APP and PS1, two susceptibility genes, which makes up for the defects of relatively single pathological changes caused by a single mutated gene and which can better simulate the pathological features and the behavior changes of AD. Similar AD animal models have been used to study the role of RNA demethylase in the pathogenesis of AD, for which triple transgenic mice were used as the AD model (Li et al., 2018). Meanwhile, a large number of studies have shown that the Aβ burden, pathological changes, and cognitive functions of 9-month-old APP/PS1 mice are notably different from those of non-transgenic mice of the same age (Xiong et al., 2011; Izco et al., 2014). In this study, we used the APP/PS1 animal model and verified its pathological changes, which are consistent with those of other studies.

A large number of studies have shown that m6A RNA methylation is related to the occurrence of tumor diseases, such as breast cancer and lung cancer (Kaklamani et al., 2011; Li et al., 2019), while relatively few studies focused on the relationship between m6A RNA methylation and the brain. In 2017, Chang and colleagues performed a transcriptome-wide methylation analysis with mouse brain samples and identified RNA m6A methylation as a new element in the region-specific gene regulatory network (Chang et al., 2017). A study by Ke et al. (2015) indicated that the m6A density peaks early in the 3′ UTR and that the brain transcripts preferentially use distal polyA sites among transcripts with alternative polyA usage in the brain, and a higher proximal m6A density was observed in the last exons. However, no study has reported the role of m6A RNA methylation in AD.

In this study, we found that there was a difference in the m6A levels between AD and control mice, and the m6A methylation genes in the AD group were related to the presynaptic membrane, the postsynaptic membrane, and the synaptic growth, altogether suggesting that m6A may indeed be involved in the occurrence of AD.

Some studies have suggested that METTL3 is related to hippocampal memory function (Zhang et al., 2018), while another study showed that in brain tissues, FTO has a wide variety of physiological and pathological functions (Li et al., 2018). In addition, one study found that METTL14, another important m6A RNA methyltransferase, is critical for the transcriptional regulation of striatum function and learning epitopes (Koranda et al., 2018). Still other studies have suggested that m6A RNA methylation is related to the development of the cerebellum (Ma et al., 2018). More interestingly, one study found that m6A methylation changes dynamically with age in the brains of mice. For that study, total RNA was isolated from mice at different ages, including 18-day-old mouse embryos, at birth, 14 days after birth, and during adulthood. Western blot analysis was then performed to detect the levels of transcription containing m6A methylation. The results showed that the expression level of m6A in mRNA was low throughout embryogenesis but increased significantly in adulthood, showing that m6A methylation has dynamic characteristics in the different stages of neural development (Meyer et al., 2012).

Among the methyltransferases, METTL3 is the core catalyst of m6A methylation. The determination of the degree of methylation between the two groups and the verification by Western blot reached the same conclusion – that the expression of METTL3 increased in the AD group.

Some studies imply that FTO is one of the main demethylases in m6A methylation, that it is abundant in the brain, and that it regulates dopaminergic signaling and adult neurogenesis (Jia et al., 2011; Hess et al., 2013; Zhao et al., 2014). In this study, Western blot and qRT-PCR showed that the expression of FTO in the AD group decreased compared to that in the control group, which was consistent with the result of increased methylation in the AD group.

Changes in synaptic function are considered to be the main mechanism of AD (Henstridge et al., 2016). At the same time, some studies have pointed out that mRNA m6A modification plays a key role in synaptic function (Weng et al., 2018; Fu et al., 2019). In our study, among the two groups of genes with changes in m6A RNA methylation levels, GO analysis results and pathway diagrams revealed several genes related to synaptic function. Differences in their expression between the two groups were further verified by qRT-PCR.

The hippocampus and the cortex are the main sections involved in AD pathogenesis, and some studies have pointed out that the development of the cerebellum is also related to m6A methylation (Ma et al., 2018). Therefore, cerebellar tissues were isolated separately in this study and compared between the two groups. The results revealed that there were no significant differences in the levels of methylation, methyltransferase, or demethylase in the cerebellum samples from the two groups, which is consistent with the fact that the main pathogenic sites of AD are located in the cerebral cortex and the hippocampus. Unfortunately, we did not compare the different brain tissues in the same group; since our mice were of the same age, a comparison of mice of different ages in the group might reveal something new. It would be interesting to analyze the m6A RNA methylation at different time points, including the time before the onset of clinical symptoms, during the development of clinical symptoms, and at the peak of misfolding protein aggregation in the animal model. Together with the results from previous studies, our results suggest that methylase and demethylase are potential targets for the treatment of AD; however, more research is needed to support these conclusions.

## Data Availability Statement

All datasets generated for this study are included in the article/supplementary material.

## Ethics Statement

The animal study was reviewed and approved by Ethical Committee for Animal Experiments of Shandong University.

## Author Contributions

YW, JB, and MH designed the research. MH performed the experiments. ZL, YX, and XL provided technical support and isolated the mouse tissue samples. XL, DW, and FL analyzed the data. YW and MH wrote the manuscript. All authors approved the manuscript for publication.

## Conflict of Interest

The authors declare that the research was conducted in the absence of any commercial or financial relationships that could be construed as a potential conflict of interest.
